# Supplementary material for: Will China’s audit of natural environmental resource promote green sustainable development? Evidence from PSM-DID analysis based on substantial and strategic pollution reduction
Source: PLoS One. 2022 Dec 13;17(12):e0278985. doi: 10.1371/journal.pone.0278985 (PMC9747048; doi:10.1371/journal.pone.0278985)
Supplement: S1 Appendix — (DOCX) [file pone.0278985.s002.docx]

**Appendix A：Descriptive Statistics.**

| **Variable** | **Mean** | **Std. Dev.** | **Min** | **Max** |
| --- | --- | --- | --- | --- |
| **Aqi** | 4.641 | 0.262 | 3.951 | 5.112 |
| **Mqi** | 133.779 | 48.084 | 50 | 297 |
| **Citysewage** | 6.544 | 0.703 | 4.819 | 7.589 |
| **Lnpgdp** | 1.767 | 0.499 | 0.419 | 2.797 |
| **Rgdp** | 0.068 | 0.044 | -0.122 | 0.273 |
| **Citypatent** | 10.859 | 1.206 | 7.606 | 12.43 |
| **Population** | 6.175 | 0.553 | 4.825 | 7.28 |
| **Popdst** | 7.867 | 7.85 | 0.06 | 58.28 |
| **Promt** | 0.225 | 0.419 | 0 | 1 |
| **SO2** | 29.832 | 18.552 | 5 | 123 |
| **PM10** | 98.321 | 34.582 | 40 | 224 |
| **PM2.5** | 59.708 | 19.806 | 22 | 129 |
| **CO** | 2.043 | 0.951 | 0.9 | 5.8 |
| **NO2** | 38.927 | 11.423 | 14 | 67 |
| **O3** | 85.859 | 16.137 | 34 | 125 |
| **Temperature** | 2.727 | 0.373 | 1.932 | 3.148 |
| **Rainfall** | 6.862 | 0.621 | 5.765 | 7.702 |
| **Humidity** | 4.263 | 0.144 | 4.007 | 4.419 |
| **Sunshine** | 7.501 | 0.318 | 6.776 | 7.909 |
| **Lncpi** | 1.533 | 0.001 | 1.531 | 1.535 |
| **Age** | 51.961 | 3.338 | 43 | 61 |
| **Edu** | 0.833 | 0.374 | 0 | 1 |
| **Tenure** | 0.235 | 0.425 | 0 | 1 |
