# Supplementary material for: Will China’s audit of natural environmental resource promote green sustainable development? Evidence from PSM-DID analysis based on substantial and strategic pollution reduction
Source: PLoS One. 2022 Dec 13;17(12):e0278985. doi: 10.1371/journal.pone.0278985 (PMC9747048; doi:10.1371/journal.pone.0278985)
Supplement: S2 Appendix — (ZIP) [file pone.0278985.s003.zip › S3 Appendix B.Table 1-7/Table 1. Baseline evaluation of green development effect..docx]

**Table 1. Baseline evaluation of green development effect.**

| **The variable**  **name** | **(1)** | **(2)** | **(3)** | **(4)** | **(5)** | **(6)** |
| --- | --- | --- | --- | --- | --- | --- |
|  | **Aqi** | **Aqi** | **Citysewage** | **Citysewage** | **Citypatent** | **Citypatent** |
| **The current effect** | -0.0566 | -0.0571 | 0.0099 | 0.0110 | -0.0083 | -0.0025 |
|  | (-1.3381) | (-1.1596) | (0.6430) | (0.8525) | (-0.4300) | (-0.1269) |
| **Delayed stage effect** | -0.1388^*^ | -0.1074^*^ | 0.0564 | 0.0379 | -0.0526 | -0.0635 |
|  | (-1.9770) | (-1.7816) | (1.0727) | (0.8375) | (-1.3093) | (-1.4570) |
| **Lnpgdp** |  | 0.2752^***^ |  | -0.2566^**^ |  | -0.3766^*^ |
|  |  | (5.2333) |  | (-2.1661) |  | (-1.6816) |
| **Popdst** |  | 0.0681^*^ |  | -0.0292^**^ |  | -0.0554^**^ |
|  |  | (1.9366) |  | (-2.1202) |  | (-2.3403) |
| **Age** |  | 0.3062^***^ |  | 0.9006^***^ |  | -0.0092^**^ |
|  |  | (9.8067) |  | (5.1559) |  | (-2.4681) |
| **Edu** |  | 0.9534^***^ |  | 0.9084^***^ |  | -0.8122^***^ |
|  |  | (5.1705) |  | (4.8811) |  | (-6.1779) |
| **Tenure** |  | -0.0094 |  | 0.2133^*^ |  | 0.8024^***^ |
|  |  | (-0.3026) |  | (1.3584) |  | (6.1283) |
| **Lncpi** |  | 18.3702^**^ |  | 7.5641^**^ |  | 1.7411^***^ |
|  |  | (2.1492) |  | (2.1496) |  | (9.2486) |
| **Population** |  | -0.2566^**^ |  | 0.8963^**^ |  | 1.4531^**^ |
|  |  | (-2.0604) |  | (2.1245) |  | (2.2442) |
| **Temperature** |  | -0.3001^**^ |  | -0.8291^***^ |  | 0.0001 |
|  |  | (-1.1011) |  | (-9.1784) |  | (1.1427) |
| **Rainfall** |  | 0.7484^***^ |  | 0.4615^***^ |  | 0.6477^***^ |
|  |  | (6.0078) |  | (6.0219) |  | (5.0757) |
| **Humidity** |  | 0.0181 |  | 0.0546^***^ |  | 0.1231^***^ |
|  |  | (0.4258) |  | (8.5318) |  | (4.6826) |
| **Sunshine** |  | 0.2201^***^ |  | 0.0665^**^ |  | 0.2490^***^ |
|  |  | (4.2512) |  | (2.4197) |  | (7.2538) |
| **_cons** | 4.5472^***^ | -80.2141^**^ | 6.5125^***^ | 36.7155^**^ | 10.7075^***^ | 11.4715^***^ |
|  | (431.2618) | (-1.9895) | (1.9×10^3^) | (2.2186) | (2.0×10^3^) | (0.0004) |
| **r2_w** | 0.5279 | 0.6232 | 0.4665 | 0.5580 | 0.9059 | 0.9160 |

Notes: *t* statistics in parentheses, ^*^ *p* < 10%, ^**^ *p* < 5%, ^***^ *p* < 1%.
