# Supplementary material for: Will China’s audit of natural environmental resource promote green sustainable development? Evidence from PSM-DID analysis based on substantial and strategic pollution reduction
Source: PLoS One. 2022 Dec 13;17(12):e0278985. doi: 10.1371/journal.pone.0278985 (PMC9747048; doi:10.1371/journal.pone.0278985)
Supplement: S2 Appendix — (ZIP) [file pone.0278985.s003.zip › S3 Appendix B.Table 1-7/Table 2. Group inspection of cities before the pilot audit..docx]

**Table 2. Group inspection of cities before the pilot audit.**

| **The variable**  **name** | **(1)** | **(2)** | **(3)** | **(4)** |
| --- | --- | --- | --- | --- |
|  | **Aqi** | **Aqi** | **Mqi** | **Mqi** |
| **The policy effect** | -0.0529 | -0.0551 | -4.2845 | 3.3527 |
|  | (-1.0961) | (-0.7327) | (-0.4020) | (0.0960) |
| **Lnpgdp** | 0.0107^**^ | 0.1507^***^ | -13.6046 | 27.9846^***^ |
|  | (2.1772) | (2.8563) | (-1.0141) | (9.6348) |
| **Popdst** |  | -0.1016^***^ |  | -13.8036^***^ |
|  |  | (-3.0296) |  | (-2.9281) |
| **Age** |  | -0.2921^***^ |  | -53.5970^**^ |
|  |  | (-3.4693) |  | (-2.2513) |
| **Edu** |  | -1.0311^***^ |  | -2.3506^***^ |
|  |  | (-2.5956) |  | (-9.0676) |
| **Tenure** |  | -0.0004^**^ |  | -0.0636 |
|  |  | (-2.5232) |  | (-0.9922) |
| **Lncpi** |  | 0.9263 |  | 1.9241^***^ |
|  |  | (0.4269) |  | (4.0236) |
| **Population** |  | 13.2195^***^ |  | 9.1103^***^ |
|  |  | (4.0023) |  | (3.0001) |
| **Temperature** |  | -0.2620 |  | 25.8102^***^ |
|  |  | (-0.9048) |  | (8.1023) |
| **Humidity** |  | -12.0331^***^ |  | 94.4018^***^ |
|  |  | (-6.0623) |  | (4.0271) |
| **Rainfall** |  | 3.0018^***^ |  | -7.4097^***^ |
|  |  | (3.0043) |  | (-4.0440) |
| **Sunshine** |  | -0.1204 |  | -41.6697^***^ |
|  |  | (-0.9403) |  | (-5.065) |
| **_cons** | 4.5691^***^ | 6.1718^*^ | 168.0509^***^ | 105.5567 |
|  | (39.4705) | (1.9879) | (6.4968) | (0.0863) |
| **r2** | 0.8125 | 0.9091 | 0.0211 | 0.7449 |

Notes: *t* statistics in parentheses, ^*^ *p* < 10%, ^**^ *p* < 5%, ^***^ *p* < 1%.
