# Supplementary material for: Will China’s audit of natural environmental resource promote green sustainable development? Evidence from PSM-DID analysis based on substantial and strategic pollution reduction
Source: PLoS One. 2022 Dec 13;17(12):e0278985. doi: 10.1371/journal.pone.0278985 (PMC9747048; doi:10.1371/journal.pone.0278985)
Supplement: S2 Appendix — (ZIP) [file pone.0278985.s003.zip › S3 Appendix B.Table 1-7/Table 3. Grouping trend test of six major air pollutants..docx]

**Table 3. Grouping trend test of six major air pollutants.**

| **The variable**  **name** | **(1)** | **(2)** | **(3)** | **(4)** | **(5)** | **(6)** |
| --- | --- | --- | --- | --- | --- | --- |
|  | **PM2.5** | **PM10** | **SO2** | **NO2** | **CO** | **O3** |
| **The policy effect** | 0.1480  (0.0347) | -3.6198  (-0.5537) | 3.7026  (1.0742) | -1.0047  (-0.3651) | 0.1255  (0.7126) | -4.3921  (-0.6074) |
| **Lnpgdp** | 18.5631^**^ | 8.3813^***^ | 14.6048^***^ | -0.2867 | 23.4542^**^ | 25.4195^***^ |
|  | (1.9890) | (4.0023) | (2.9193) | (-0.7214) | (2.5066) | (2.6054) |
| **Popdst** | 3.5518^***^ | 0.1556^***^ | 0.6419^**^ | 0.0334 | 2.1500^***^ | -0.2474 |
|  | (4.3593) | (4.0001) | (2.2917) | (1.2524) | (4.2580) | (-0.4119) |
| **Age** | 1.8542^***^ | -0.9615^*^ | 0.4123^***^ | 0.0306^***^ | 0.2420^***^ | 0.5606^**^ |
|  | (6.0021) | (-1.6868) | (5.0091) | (3.7367) | (3.0082) | (3.0144) |
| **Edu** | -4.6529^***^ | -10.5028^***^ | -0.7700 | -0.2328^*^ | -3.3428^***^ | 10.8036^*^ |
|  | (-6.1256) | (-6.0650) | (-0.5238) | (-1.8977) | (-3.0059) | (1.8927) |
| **Tenure** | 2.6005^***^ | -1.0654^***^ | 0.8388^*^ | 0.1210^**^ | 2.3753 | 2.0447^***^ |
|  | (4.0007) | (-6.0019) | (0.0347) | (2.3922) | (1.1097) | (5.0055) |
| **Lncpi** | -3.0×10^3*^ | 558.6731^***^ | -1.4×10^2^ | -20.3887^***^ | -3.5×10^3^ | 3.4×10^3***^ |
|  | (-0.0009) | (0.0005) | (-0.2023) | (-0.0006) | (-1.3957) | (0.0740) |
| **Population** | 0.9892^***^ | 6.2382^***^ | 6.3415^**^ | -0.0374 | 1.3915 | 6.0119^***^ |
|  | (6.0090) | (6.0084) | (2.3408) | (-0.1249) | (0.2949) | (8.0028) |
| **Temperature** | 29.8704^**^ | 4.5422^***^ | -8.4289^***^ | -0.1826^**^ | 21.1819^***^ | 26.2144 |
|  | (2.5223) | (7.0058) | (-4.0040) | (-0.0098) | (3.9914) | (1.6044) |
| **Rainfall** | -45.4872^***^ | -16.4306^*^ | -9.0856^***^ | -0.4813^*^ | -28.3557^***^ | -12.9488 |
|  | (-3.4253) | (-1.9288) | (-2.8317) | (-1.7120) | (-4.1337) | (-1.3472) |
| **Humidity** | -19.9851^***^ | -47.2900^***^ | -21.8089 | -1.8879^*^ | 10.8801^***^ | -27.5731^***^ |
|  | (-5.0027) | (-3.0077) | (-1.5467) | (-1.9460) | (7.2063) | (-4.0609) |
| **Sunshine** | -16.4576^*^ | -8.9974^**^ | -7.1881^*^ | -8.9104^***^ | -12.3238^**^ | -0.5583 |
|  | (-1.8672) | (-2.0851) | (-1.7901) | (-3.0061) | (-1.9775) | (-0.0600) |
| **_cons** | 5.2×10^3***^ | -4.7×10^2***^ | 390.6422^***^ | 44.1609 | 5.6×10^3^ | -5.2×10^3***^ |
|  | (6.1306) | (-7.0046) | (13.0003) | (0.5201) | (1.4350) | (-223.0002) |
| **r2_w** | 0.6973 | 0.6349 | 0.6339 | 0.7245 | 0.5864 | 0.5336 |

Notes: *t* statistics in parentheses, ^*^ *p* < 10%, ^**^ *p* < 5%, ^***^ *p* < 1%.
