# Supplementary material for: Will China’s audit of natural environmental resource promote green sustainable development? Evidence from PSM-DID analysis based on substantial and strategic pollution reduction
Source: PLoS One. 2022 Dec 13;17(12):e0278985. doi: 10.1371/journal.pone.0278985 (PMC9747048; doi:10.1371/journal.pone.0278985)
Supplement: S2 Appendix — (ZIP) [file pone.0278985.s003.zip › S3 Appendix B.Table 1-7/Table 4. Placebo test results..docx]

**Table 4. Placebo test results.**

| **The variable**  **name** | **(1)** | **(2)** | **(3)** | **(4)** |
| --- | --- | --- | --- | --- |
|  | **Aqi** | **Aqi** | **Mqi** | **Mqi** |
| **The current effect** | -6.5046 | -0.2283 | -34.9693^*^ | -1.8798 |
|  | (-1.0738) | (-0.0286) | (-1.8868) | (-0.0987) |
| **Delayed stage effect** | 2.1786 | -0.8542 | 17.5310 | -2.7880 |
|  | (0.3390) | (-0.1050) | (1.4050) | (-0.1771) |
| **Lnpgdp** |  | 9.4748^***^ |  | -21.4160^***^ |
|  |  | (9.0077) |  | (-8.0099) |
| **Popdst** |  | 6.8395^**^ |  | 16.9232^*^ |
|  |  | (2.1740) |  | (1.7370) |
| **Age** |  | -5.0672^***^ |  | -7.1595^**^ |
|  |  | (-3.3110) |  | (-2.5974) |
| **Edu** |  | -5.4963^***^ |  | -34.4800^**^ |
|  |  | (-2.8710) |  | (-2.2205) |
| **Tenure** |  | 0.8784^***^ |  | -3.1629^***^ |
|  |  | (5.4129) |  | (-5.2933) |
| **Lncpi** |  | -1.9×10^2***^ |  | -1.4×10^3**^ |
|  |  | (-2.5413) |  | (-2.2625) |
| **Population** |  | -94.6750 |  | -3.6×10^2^ |
|  |  | (-1.0930) |  | (-1.3846) |
| **Temperature** |  | 0.4710^**^ |  | 7.1855^**^ |
|  |  | (2.0450) |  | (2.2285) |
| **Rainfall** |  | -0.7998^**^ |  | -3.9922^***^ |
|  |  | (-2.2540) |  | (-8.4361) |
| **Humidity** |  | -12.0332^***^ |  | -14.0013^***^ |
|  |  | (-0.0013) |  | (-0.0025) |
| **Sunshine** |  | -0.0377^***^ |  | -0.1837^***^ |
|  |  | (-2.6417) |  | (-4.0313) |
| **_cons** | 91.9646^***^ | 1.5×10^3^ | 141.0751^***^ | 8.9×10^3^ |
|  | (125.9169) | (0.9370) | (51.0046) | (1.4870) |
| **r2** | 0.6389 | 0.5512 | 0.7209 | 0.5211 |

Notes: *t* statistics in parentheses, ^*^ *p* < 10%, ^**^ *p* < 5%, ^***^ *p* < 1%.
