# Supplementary material for: Will China’s audit of natural environmental resource promote green sustainable development? Evidence from PSM-DID analysis based on substantial and strategic pollution reduction
Source: PLoS One. 2022 Dec 13;17(12):e0278985. doi: 10.1371/journal.pone.0278985 (PMC9747048; doi:10.1371/journal.pone.0278985)
Supplement: S2 Appendix — (ZIP) [file pone.0278985.s003.zip › S3 Appendix B.Table 1-7/Table 5. Estimation of THE PSW-DID model..docx]

**Table 5. Estimation of THE PSW-DID model.**

| **The variable**  **name** | **(1)** | **(2)** | **(3)** | **(4)** |
| --- | --- | --- | --- | --- |
|  | **Aqi** | **Mqi** | **Citysewage** | **Citypatent** |
| **The current effect** | 0.0605 | -32.9898^*^ | -0.0326 | 0.0687 |
|  | (1.0481) | (-1.9290) | (-1.0169) | (1.4877) |
| **Delayed stage effect** | -0.2290^*^ | -59.0879^***^ | 0.0027 | -0.0761 |
|  | (-1.9738) | (-4.2056) | (0.0424) | (-0.7617) |
| **Lnpgdp** | -0.4215^***^ | -9.6118^***^ | 0.6977^***^ | 0.2590^***^ |
|  | (-3.5928) | (-4.0697) | (2.7222) | (4.2535) |
| **Popdst** | 0.1202^***^ | 16.6264 | -0.2289^*^ | -0.0195^**^ |
|  | (3.5367) | (0.4599) | (-2.1037) | (-2.0655) |
| **Age** | 0.0443^***^ | 8.6318^***^ | 0.0115 | 0.0495^***^ |
|  | (3.0744) | (2.6301) | (0.7583) | (3.0516) |
| **Edu** | -0.5428^***^ | -48.3532 | 0.0559^***^ | 0.1358^**^ |
|  | (-3.5613) | (-0.9560) | (6.3870) | (2.2249) |
| **Tenure** | -0.0502 | 3.7431^***^ | -0.0258^**^ | 0.0028^***^ |
|  | (-0.8690) | (3.3232) | (-1.9886) | (3.0716) |
| **Lncpi** | -24.8530^***^ | -1.7×10^3^ | 2.4785^***^ | 13.7306^**^ |
|  | (-6.4878) | (-0.7218) | (3.3986) | (1.9487) |
| **Population** | -3.3479^*^ | -1.5×10^2^ | 5.7337^***^ | 0.1742^**^ |
|  | (-1.8739) | (-0.2827) | (6.3125) | (2.0580) |
| **Temperature** | 1.6730^*^ | 45.5612 | -0.1255 | 0.2308^*^ |
|  | (1.7455) | (0.3167) | (-0.3421) | (2.1092) |
| **Humidity** | 3.7248^**^ | -125.6758^***^ | 0.3310 | 0.5002 |
|  | (2.7547) | (-3.7088) | (0.6238) | (0.2710) |
| **Rainfall** | -1.2965^**^ | 15.5638 | -0.0969^***^ | 0.1045 |
|  | (-2.4763) | (0.2105) | (-3.4794) | (0.1315) |
| **Sunshine** | -0.2910^*^ | 68.8157^***^ | 0.4018^***^ | -0.6944 |
|  | (-2.0605) | (3.5896) | (3.4160) | (-0.8264) |
| **_cons** | -1.1×10^2^ | 7.0×10^3^ | -43.0178 | 76.8525 |
|  | (-1.2244) | (0.5503) | (-1.2461) | (0.6410) |
| **r2_w** | 0.8608 | 0.7982 | 0.8699 | 0.8978 |

Notes: *t* statistics in parentheses, ^*^ *p* < 10%, ^**^ *p* < 5%, ^***^ *p* < 1%.
