# Supplementary material for: Will China’s audit of natural environmental resource promote green sustainable development? Evidence from PSM-DID analysis based on substantial and strategic pollution reduction
Source: PLoS One. 2022 Dec 13;17(12):e0278985. doi: 10.1371/journal.pone.0278985 (PMC9747048; doi:10.1371/journal.pone.0278985)
Supplement: S2 Appendix — (ZIP) [file pone.0278985.s003.zip › S3 Appendix B.Table 1-7/Table 6. Discussion of the mechanism of economic performance and manpower level..docx]

**Table 6. Discussion of the mechanism of economic performance and manpower level.**

| **The variable**  **name** | **(1)** | **(2)** | **(3)** | **(4)** |
| --- | --- | --- | --- | --- |
|  | **Gdp** | **Lnpgdp** | **Popdst** | **Population** |
| **The current effect** | 0.1946^***^ | 0.1480^**^ | 0.0468 | 0.0052 |
|  | (1.7×10^3^) | (2.3311) | (0.3440) | (0.8733) |
| **Delayed stage effect** | 0.3410^***^ | 0.3154^**^ | 0.1888 | 0.0093 |
|  | (2.9×10^3^) | (2.2239) | (0.8774) | (1.0358) |
| **Lnpgdp** |  |  | 0.5195^***^ | 7.3355^***^ |
|  |  |  | (4.2×10^3^) | (6.2940) |
| **Popdst** | 1.8916^***^ | 0.0381^***^ |  |  |
|  | (4.6×10^3^) | (3.3682) |  |  |
| **Age** | 0.0392^***^ | 0.0188^***^ | -0.0376^***^ | -0.0015^*^ |
|  | (3.4×10^3^) | (3.5612) | (-4.4827) | (-1.8414) |
| **Edu** | 0.0011^***^ | 0.0658 | -0.2648^*^ | -0.0092^*^ |
|  | (21.6586) | (0.8329) | (-1.7472) | (-1.7481) |
| **Tenure** | 0.0541^***^ | 0.0374^***^ | -0.4128 | -0.0114 |
|  | (1.5×10^3^) | (3.8670) | (-1.2697) | (-1.0581) |
| **Lncpi** | 0.4743^***^ | 10.1076^***^ | 15.7632^***^ | 0.3019^***^ |
|  | (33.9770) | (8.0734) | (3.7079) | (6.4025) |
| **Population** | 4.9704^***^ | 1.7464^***^ |  |  |
|  | (9.5×10^2^) | (6.6130) |  |  |
| **Temperature** | -0.3187^***^ | -0.1575 | 0.3280^***^ | 0.0072 |
|  | (-2.1×10^4^) | (-1.3211) | (3.6623) | (0.5169) |
| **Humidity** | -0.1078^***^ | -0.0312 | -0.4199^**^ | -0.0123^**^ |
|  | (-1.1×10^3^) | (-0.5235) | (-2.1053) | (-2.4041) |
| **Rainfall** | 0.0014^***^ | 0.4725^**^ | 0.0022^**^ | 0.6001^**^ |
|  | (1.4×10^4^) | (2.1847) | (2.5544) | (2.4353) |
| **Sunshine** | 0.1245^***^ | -0.0726 | 0.5300 | 0.0069 |
|  | (1.5×10^3^) | (-1.0858) | (1.3477) | (0.4973) |
| **_cons** | 50.5926^***^ | -65.3584 | -53.5747 | 4.7174 |
|  | (501.6961) | (-1.3786) | (-0.5403) | (1.3893) |
| **r2_w** | 1.0000 | 0.9142 | 0.3286 | 0.1228 |

Notes: *t* statistics in parentheses, ^*^ *p* < 10%, ^**^ *p* < 5%, ^***^ *p* < 1%.
