# Supplementary material for: Will China’s audit of natural environmental resource promote green sustainable development? Evidence from PSM-DID analysis based on substantial and strategic pollution reduction
Source: PLoS One. 2022 Dec 13;17(12):e0278985. doi: 10.1371/journal.pone.0278985 (PMC9747048; doi:10.1371/journal.pone.0278985)
Supplement: S2 Appendix — (ZIP) [file pone.0278985.s003.zip › S3 Appendix B.Table 1-7/Table 7. Discussion on leading cadres' promotion assessment and manpower level..docx]

**Table 7. Discussion on leading cadres' promotion assessment and manpower level.**

| **The variable**  **name** | **(1)** | **(2)** | **(3)** | **(4)** | **(5)** | **(6)** | **(7)** |
| --- | --- | --- | --- | --- | --- | --- | --- |
|  | **Promt** | **Promt** | **Promt** | **Promt** | **Aqi** | **Citysewage** | **Rgdp** |
| **Aqi** | -0.0322^*^ |  |  |  |  |  |  |
|  | (-1.7439) |  |  |  |  |  |  |
| **Citysewage** |  | -0.2057 |  |  |  |  |  |
|  |  | (-0.7183) |  |  |  |  |  |
| **Rgdp** |  |  | 3600* |  |  |  |  |
|  |  |  | (1.7991) |  |  |  |  |
| **Population** |  |  |  | -0.6566 | 0.1535^***^ | 0.1742^*^ | 0.3593^***^ |
|  |  |  |  | (-1.5503) | (2.8638) | (1.6561) | (3.6000) |
| **Lnpgdp** | 0.0700^***^ | 0.0238^***^ | 4.9040^**^ | 0.2480^***^ | 0.1099^***^ | 0.1344^***^ | 0.1562^**^ |
|  | (3.3297) | (3.0439) | (1.9824) | (6.5050) | (4.5725) | (3.7867) | (1.9719) |
| **Popdst** |  | 0.0257 | 0.1083^**^ |  | -0.0027 | 0.0067 | -0.0232^***^ |
|  |  | (0.8774) | (2.2807) |  | (-0.7912) | (1.2983) | (-4.3774) |
| **Age** | -0.0070^**^ | -0.0091^***^ | -0.0375^**^ | -0.3068^***^ | 0.0018 | 0.0006^***^ | -0.0009 |
|  | (-2.0852) | (-4.1534) | (-2.3493) | (-3.1177) | (0.2912) | (3.2020) | (-0.2684) |
| **Edu** | 0.4518^***^ | 0.5166^**^ | 0.9764^***^ | 0.5331^**^ | 0.0167 | 0.6096^***^ | 0.0099^***^ |
|  | (6.7927) | (1.9338) | (4.4206) | (1.9760) | (0.3823) | (4.4618) | (3.4184) |
| **Tenure** | 0.2637^***^ | 0.1327 | 0.0836^*^ | 0.1382 | -0.0236^***^ | 2.0190^***^ | 0.3297^***^ |
|  | (2.5282) | (0.3189) | (1.8193) | (0.3326) | (-3.7944) | (3.6425) | (3.0516) |
| **Lncpi** | 1.3691^***^ | 61.7774^***^ | 7.6609^***^ | 54.2795^***^ | 0.3863^***^ | -7.7208^***^ | 2.5460^***^ |
|  | (3.2665) | (13.9152) | (26.3320) | (16.7975) | (-9.0649) | (-3.1972) | (8.9236) |
| **Temperature** | 0.8532 | -0.6918 | 0.9623^**^ | -0.4849 | -0.0168^***^ | 3.0023^***^ | 0.0001 |
|  | (0.7891) | (-0.7205) | (1.9906) | (-0.5303) | (-3.3030) | (9.1158) | (0.0028) |
| **Humidity** | 0.0319 | 0.2660 | 3.5201 | 0.6003^***^ | -0.0063^***^ | 1.3690 | 2.9306 |
|  | (0.0031) | (0.0930) | (0.0206) | (3.0031) | (-5.0635) | (0.0616) | (0.2631) |
| **Rainfall** | 0.0019^**^ | 0.0906 | 0.0019 | 0.7011 | 5.1301^***^ | -0.6319^***^ | 0.0002^**^ |
|  | (2.0139) | (0.6590) | (0.9908) | (1.5214) | (6.9865) | (-3.2494) | (2.5485) |
| **Sunshine** | 1.3026 | 0.2746 | 2.0102 | 0.2842 | -0.1957^***^ | 1.0049^***^ | 0.0192^***^ |
|  | (0.0360) | (0.5284) | (0.1362) | (0.5447) | (-5.1149) | (3.3269) | (4.1208) |
| **_cons** | 2.9367 | 288.4970 | 1.9933 | 254.1586 | 1.7573 | 41.2278^***^ | 11.5723 |
|  | (0.5221) | (0.9223) | (0.2474) | (0.8057) | (0.0637) | (3.6776) | (0.9056) |
| **r2_w** | 0.6921 | 0.7103 | 0.5662 | 0.7209 | 0.5276 | 0.5185 | 0.5584 |

Notes: *t* statistics in parentheses, ^*^ *p* < 10%, ^**^ *p* < 5%, ^***^ *p* < 1%.
